# Suppression of type III effector secretion by polymers

**DOI:** 10.1098/rsob.130133

**Published:** 2013-12

**Authors:** Takashi Ohgita, Naoki Hayashi, Naomasa Gotoh, Kentaro Kogure

**Affiliations:** 1 Department of Biophysical Chemistry, Kyoto Pharmaceutical University, Misasagi-Nakauchicho 5, Yamashina-ku, Kyoto 607-8414, Japan; 2 Department of Microbiology and Infection Control Science, Kyoto Pharmaceutical University, Misasagi-Nakauchicho 5, Yamashina-ku, Kyoto 607-8414, Japan

**Keywords:** type III secretion, polymer, *Pseudomonas aeruginosa*, PEG, alginate, mucin

## Abstract

Bacteria secrete effector proteins required for successful infection and expression of toxicity into host cells. The type III secretion apparatus is involved in these processes. Previously, we showed that the viscous polymer polyethylene glycol (PEG) 8000 suppressed effector secretion by *Pseudomonas aeruginosa*. We thus considered that other viscous polymers might also suppress secretion. We initially showed that PEG200 (formed from the same monomer (ethylene glycol) as PEG8000, but which forms solutions of lower viscosity than the latter compound) did not decrease effector secretion. By contrast, alginate, a high-viscous polymer formed from mannuronic and guluronic acid, unlike PEG8000, effectively inhibited secretion. The effectiveness of PEG8000 and alginate in this regard was closely associated with polymer viscosity, but the nature of viscosity dependence differed between the two polymers. Moreover, not only the natural polymer alginate, but also mucin, which protects against infection, suppressed secretion. We thus confirmed that polymer viscosity contributes to the suppression of effector secretion, but other factors (e.g. electrostatic interaction) may also be involved. Moreover, the results suggest that regulation of bacterial secretion by polymers may occur naturally via the action of components of biofilm or mucin layer.

## 2. Introduction

The bacterial type III secretion apparatus (T3SA) is a needle-like molecular assemblage, very similar in structure to the bacterial flagellum [1]. When bacteria expressing T3SA (e.g. *Pseudomonas aeruginosa* and *Shigella flexneri*) contact their host cells, T3SA is activated, and the tip binds to the translocation pore (constructed by translocator proteins secreted in advance via T3SA) at the host cell membrane. Then, bacterial proteins (termed effectors) are injected directly into the host cytoplasm via T3SA through the translocation pore. Effectors mediate bacterial infection and toxicity by performing specific functions (e.g. cytoskeletal rearrangement or induction of apoptosis) in the host cell [1,2].

In a previous study, we developed a system whereby T3SA rotation could be observed microscopically; we added a microbead to the tip of T3SA. We found, first, that T3SA rotates in a manner similar to the bacterial flagellum when effectors are secreted. Second, rotation was inhibited by the addition of a protonophore (CCCP) that reduces the proton-motive force. Finally, the highly viscous polymer polyethylene glycol (PEG) 8000 inhibited both the rotation of and effector secretion by T3SA. The results suggested that effector secretion by T3SA involved the proton-motive force-dependent rotation of the T3SA needle [3].

It is known that motility of molecular assemblages (e.g. the flagellum or F1-ATPase) is usually required for functionality, and physico-chemical inhibition of motility via induction of viscous resistance inhibits functions [4,5]. Therefore, we predicted that highly viscous PEG8000 suppressed *P. aeruginosa* effector secretion by T3SA via physico-chemical inhibition of T3SA rotation. However, it is also known that hydrogen bonding and van der Waals interactions play major roles in the interaction of PEGs with living cells [6,7]. Therefore, it was possible that PEG8000 inhibited effector secretion by T3SA not only via a viscosity-mediated physico-chemical inhibition of T3SA rotation but also via another mechanism. Therefore, in this study, we explored the effects of various polymers on effector secretion by and rotation of T3SA to identify the relevant characteristics of polymers suppressing effector secretion. The viscosities of solutions of the various polymers were measured, and the relationship between viscosity and inhibitory activity was evaluated. Based on the results, the inhibitory actions of various polymers are discussed.

## 3. Material and methods

### 3.1. Materials

The components of Luria–Bertani (LB) medium were purchased from Funakoshi Co. (Tokyo, Japan). The calcium chelator ethylene glycol tetra-acetic acid (EGTA) was the product of Dojindo Molecular Technologies (Kumamoto, Japan). PEG8000 was purchased from MP Biomedicals (Tokyo, Japan). PEG200, alginic acid (sodium salt) and bovine submaxillary gland mucin were the products of Sigma-Aldrich (St Louis, MO). All other chemicals were of reagent grade and were used without further purification.

### 3.2. Bacterial strains

*Pseudomonas aeruginosa* PAO1 has been completely sequenced [8]. The PAO1 Strep strain was constructed using the suicide vector pEX18Tc and plasmid pME6032 as described previously [3]. Bacteria were grown in LB broth or on LB agar plates at 37°C.

### 3.3. Measurement of effector secretion by Western blotting

T3SA-mediated effector (ExoT) secretion was assayed using a previously described method [9]. *Pseudomonas aeruginosa* PAO1 was diluted 1 : 300 from an overnight culture and incubated for 2.5 h at 37°C in high-salt LB broth (LB broth with 200 mM NaCl, 0.5 mM CaCl_2_ and 10 mM MgCl_2_) supplemented with 5 mM EGTA. The culture was next divided into aliquots of 1.5 ml and each aliquot was centrifuged at 9000 r.p.m. for 3 min at 4°C. Supernatants were collected and the pellets resuspended in 1 ml amounts of prewarmed high-salt LB broth with 5 mM EGTA and test materials. The cultures were incubated for an additional 2.5 h and again centrifuged as described above. Supernatants were collected and the pellets resuspended in PBS; OD_600_ values of the suspensions were measured. Proteins in the first and second supernatants were precipitated using trichloroacetic acid/acetone (samples treated with PEGs) or methanol/chloroform (samples treated with alginate or mucin). The supernatant proteins were resuspended, at equivalent OD_600_ values, in 1× SDS sample buffer. All samples were boiled for 5 min at 95°C and analysed using SDS-PAGE followed by Western blotting, employing anti-ExoT rabbit IgG and anti-rabbit goat IgG-HRP as primary and secondary antibodies, respectively. Chemiluminescence-mediated detection employed ECL Prime Western Blotting Detection reagents (GE Healthcare UK, Little Chalfont, UK). ExoT band intensities were quantified using NIH ImageJ software. Band intensities in the second supernatants were normalized to those in the first supernatants, and the values shown are relative to those of controls (set at 1.0).

### 3.4. Enumeration of viable bacteria

Viable bacteria were enumerated by colony counting. PAO1 was diluted 1 : 300 from an overnight culture, and after incubation at 37°C for 5 h, appropriate dilutions were spread onto LB agar plates. Bacterial colony numbers were counted after overnight incubation at 37°C, and the numbers of colony-forming units (CFU) per millilitre were calculated. The values shown are relative to those of controls (set at 1.0).

### 3.5. T3SA rotation assay

A flow cell permitting microscopic observation was constructed using a poly-l-lysine-coated glass slide (75 × 75 mm^2^; Matsunami Glass Ind., Osaka, Japan), a coverslip (25 × 60 mm^2^; Matsunami Glass Ind.) and double-sided tape. Overnight cultures of PAO1 Strep were diluted 1 : 300 in high-salt LB broth supplemented with 5 mM EGTA and 125 μM IPTG. After incubation at 37°C for 5 h, cultures were diluted to OD_600_ = 0.1 and transferred to the flow cell. Incubation at room temperature for 30 min followed; the flow cell was next washed with PBS, and a 3% (w/v) BSA solution was applied. After incubation for 30 min, the flow cell was washed once more and streptavidin-coated fluorescent microbeads (Streptavidin Fluoresbrite YG Microspheres, 1.0 μm in diameter; Polyscience, Warrington, PA) (a 1 : 2000 suspension) were applied. After incubation for 30 min, the flow cell was washed and the flow cell buffer was replaced with high-salt LB broth supplemented with 5 mM EGTA. After incubation at 37°C for 30 min, microbead motion was observed using fluorescence microscopy (IX-71; Olympus, Tokyo, Japan).

### 3.6. Motion-track analysis of T3SA rotation

Motion-track analysis of T3SA rotation was performed using Adobe After Effects CS3 software (Adobe Systems, San Jose, CA). Numeric data on T3SA motion were extracted and used to generate tracking graphs with the aid of Microsoft Excel 2007 (Microsoft, Redmond, WA).

### 3.7. Viscosity and osmotic pressure of polymer solutions

The viscosities of polymer solutions were measured at 35°C using an SV-10 vibroviscometer (A&D Company, Tokyo, Japan). The osmolarity of polymer solutions was measured at room temperature using a VAPRO Vapor Pressure Osmometer 5600 (Wescor, South Logan, UT).

### 3.8. Analysis of *exoT*-gene expression level by real-time PCR

*Pseudomonas aeruginosa* PAO1 was diluted 1 : 300 from an overnight culture and incubated for 2.5 h at 37°C in high-salt LB broth supplemented with 5 mM EGTA. The culture was centrifuged at 9000 r.p.m. for 3 min at 4°C, and resuspended in 3 ml amounts of prewarmed high-salt LB broth with 5 mM EGTA and 20% PEG8000. The cultures were incubated for an additional 2.5 h and again centrifuged as described above. Total RNA was isolated from the bacterial cells with RNeasy Plus mini kit (Qiagen, Tokyo, Japan) using the method recommended by the manufacturer. cDNA was synthesized using Random Primer (nonadeoxyribonucleotide mixture; pd (N)_9_) (Takara Bio, Shiga, Japan) and Super Script III Reverse Transcriptase (Takara Bio). Then, real-time PCR was performed using a StepOne real-time PCR System (Applied Biosystems, Foster City, CA) and SYBR *Premix Ex Taq* II (Tli RNaseH Plus) (Takara Bio) with the primers for *exoT* (5′-TCTCAGCAGAACCCGTCTTTCGTGGCTGAG-3′ and 5′-AGCATCATCTGCTTGATCTCGGCGGCAGAG-3′) and *gyrB* (5′-TGCTGAAGGGGCTGGATGCCGTACGCAAGC-3′ and 5′-TATCCACCGGAATACCGCGTCCATTGTCGC-3′) [10]. *gyrB* was used as internal control. Relative mRNA expression was determined using the 2^−*ΔΔ*Ct^ method.

## 4. Results

### 4.1. Neither modification of T3SA tip by addition of the Strep tag II peptide nor peptide interaction with microbeads is required for the suppression of effector secretion by PEG8000

**Figure 1.**
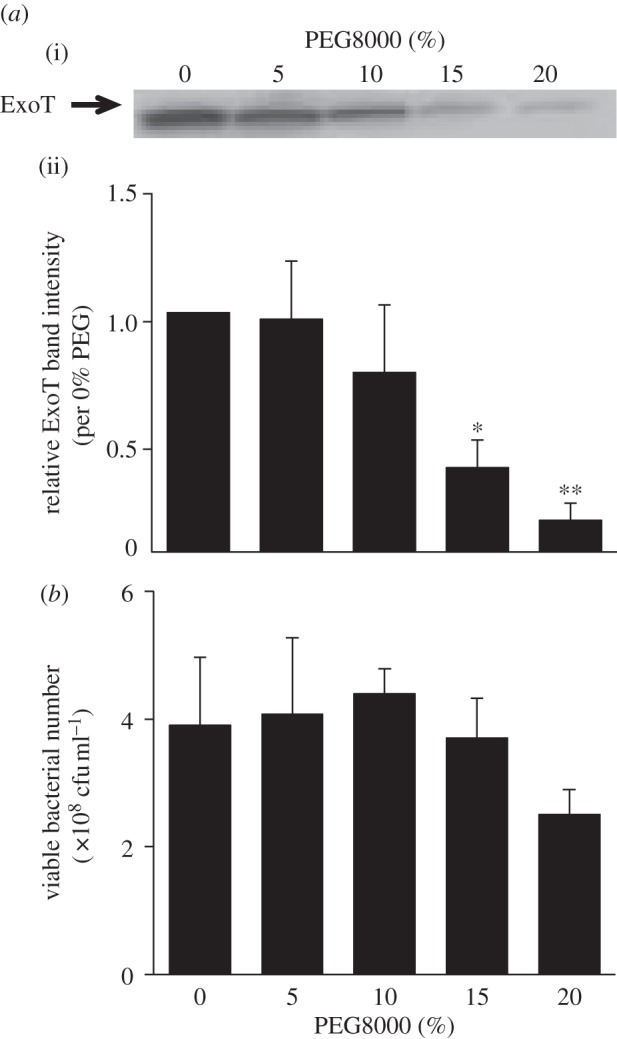
Effect of PEG8000 on secretion of the effector ExoT by WT PAO1. (*a*(i)) Western blotting of ExoT protein in the supernatants of WT PAO1 cultures exposed to different concentrations of PEG8000. After 2.5 h of T3SA activation in secretion medium, PAO1 was cultured under various conditions for 2.5 h at 37°C. The levels of ExoT in culture supernatants were analysed by Western blotting using an anti-ExoT polyclonal antibody. (*a*(ii)) The effect of PEG8000 on secretion of ExoT as shown in (*a*(i)) above. Band intensities were measured using ImageJ software. The graph shows intensities relative to that of the control (set at 1.0 at 0% (w/v) PEG8000). Average values ± s.d. (*n* = 3) are shown. *p < 0.05, **p < 0.01. (*b*) Effect of PEG8000 on bacterial viability as measured by colony counting. Average values ± s.d. (*n* = 3) are shown. No significant differences were seen in bacterial viability between 0% and 5–20% PEG8000-added samples (*p* < 0.05 versus 0% PEG8000).

In our previous study, we reported that the hydrophilic polymer PEG8000 inhibited both T3SA rotation and effector secretion by a *P. aeruginosa* PAO1 Strep strain in which the tip of T3SA was modified by addition of a Strep tagII peptide that was further bound to microbeads [3]. To confirm that such suppression of effector secretion was independent of the presence of the Strep tagII peptide and the microbeads, we explored suppression by PEG8000 of secretion by PAO1 wild-type (WT). PEG8000 inhibited effector secretion by PAO1 WT in a dose-dependent manner, especially when the PEG8000 level was above 15% (w/v) (figure 1*a*). Bacterial viability was not affected when the PEG8000 level was 20% (w/v) or less (figure 1*b*). Expression level of *exoT*-gene was activated and not suppressed when 20% (w/v) PEG8000 was added (see electronic supplementary material, figure S1). These data confirmed that PEG8000 suppressed effector secretion when T3SA was unmodified and that such suppression by PEG8000 was concentration-dependent. Moreover, it was also indicated that the suppression on effector secretion by PEG8000 was not caused by the suppression of transcriptional expression of effector genes.

### 4.2. Contribution of properties derived from the component of polyethylene glycol to the suppression of effector secretion

**Figure 2.**
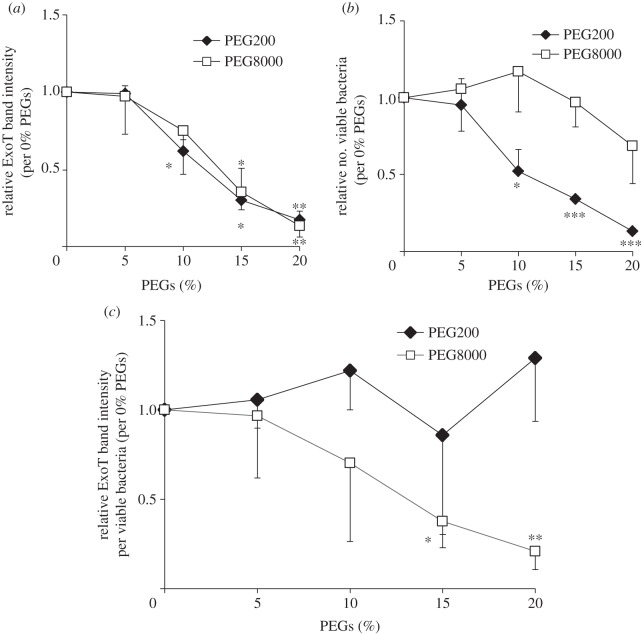
Effect of PEG8000 and PEG200 on secretion of the effector ExoT by WT PAO1. (*a*) Western blot analysis of ExoT protein levels in supernatants of PAO1 cultured in the presence of several concentrations of PEG200 and PEG8000. After the activation of PAO1 T3SA expression via culture in secretion medium for 2.5 h, bacteria were pelleted by centrifugation, then resuspended with the same medium supplemented with various concentrations of PEGs and incubated for a further 2.5 h at 37°C. The levels of ExoT in culture supernatants were analysed by Western blotting using an anti-ExoT polyclonal antibody. ExoT band intensities were measured using ImageJ software. The values shown are relative to those of the control (0% PEGs) (the control values were set to 1.0). Average values ± s.d. (*n* = 3) are shown. *p < 0.05, **p < 0.01. (*b*) Effect of PEG200 and PEG8000 on bacterial viability (CFU ml^−1^) as measured by colony counting. The values shown are relative to those of the control (0% PEGs) (the control values were set to 1.0). Average values ± s.d. (*n* = 3) are shown. *p < 0.05, ***p < 0.001. (*c*) Effects of the PEGs on the level of ExoT secreted per viable bacterium. Values were calculated by dividing the relative ExoT band intensities (*a*) by the relative numbers of viable bacteria (*b*). Average values ± s.d. (*n* = 3) are shown. *p < 0.05, **p < 0.01.

PEG has been reported to interact with living cells via hydrogen bonding and/or van der Waals interactions attributable to actions of the monomeric component, ethylene glycol [6,7]. It was thus possible that intrinsic properties of ethylene glycol might contribute to the suppression of effector secretion by PEG8000. To explore this possibility, we examined the effect of PEG200 on effector secretion by PAO1 WT. PEG200 reduced bacterial viability when present at over 10% (v/v), but PEG8000 did not affect viability when present below 20% (w/v) (figure 2*b*). We observed that osmotic pressure correlated highly with bacterial viability (see electronic supplementary material, figure S3), and hence suggested that osmotic pressure is the major mechanism by which PEG200 exerts toxicity. Effector secretion suppressed by PEG8000 recovered strongly when PEG8000 was removed by washing with high-salt LB broth (see electronic supplementary material, figure S2). These results suggest that PEG8000 does not influence viability. We normalized the suppressor effects of the two PEGs on effector secretion to the number of viable bacteria in order to exclude PEG200 toxicity (figure 2*c*). The level of effector secreted per viable bacterium was not affected by PEG200, although PEG8000 significantly decreased secretion (figure 2*c*). Although the monomeric components of PEG200 and PEG8000 are identical, the viscosities of the two materials (which are dependent on molecular size) differ. Specifically, the viscosities of 20% PEG8000 and PEG200 were 17.7 and 1.22 mPa·s, respectively (see electronic supplementary material, table S1). The results suggest that intrinsic properties of the monomer do not contribute to the suppression of effector secretion, which is instead attributable to the increase in viscosity mediated by dissolved PEG8000.

### 4.3. Contribution of inhibition of T3SA rotation to the suppression of effector secretion by PEG8000

**Figure 3.**
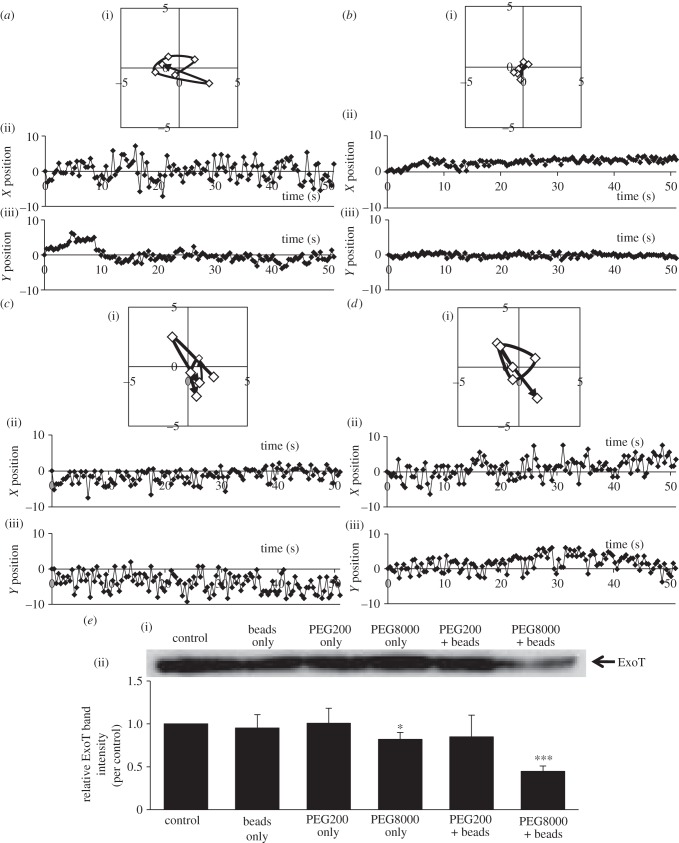
Effects of PEG8000 and PEG200 on T3SA rotation and effector secretion by PAO1 Strep. (*a–d*) Motion tracks of single microbeads that displayed typical behaviour are shown. Motion-track analysis was performed using Adobe After Effects CS3. (i) Time-dependent changes in bead tracks (7.5 points s^−1^). The black arrow indicates the direction of bead motion. (ii)–(iii) Time-dependent changes in the (ii) *X* and (iii) *Y* positions of the beads (2.5 points s^−1^). (*a*) Before and (*b*) after PEG8000 treatment. (*c*) Before and (*d*) after PEG200 treatment. (*e*(i)) Western blot analysis of ExoT protein levels in supernatants of PAO1 Strep cultured under different conditions. After activation of T3SA by 2.5 h of culture in secretion medium, PAO1 Strep was cultured under various conditions for a further 2.5 h at 37°C. The levels of ExoT in culture supernatants were analysed by Western blotting using an anti-ExoT polyclonal antibody. (ii) Effects of PEG8000 and PEG200 on ExoT secretion by T3SA tagged with microbeads shown in (*e*(i)). Band intensities were measured using ImageJ software. The graph shows relative intensity values (i.e. the control value was set to 1.0). Average values ± s.d. (*n* = 3) are shown. *p < 0.05, ***p < 0.001.

To explore a possible association between the inhibition of T3SA rotation and suppression of effector secretion, we examined the effects of PEG200 and PEG8000 on rotation evaluated using the system described in our previous study [3]. T3SA rotation was microscopically observed by tagging the tip of T3SA of PAO1 Strep with a Strep tagII peptide binding a microbead. PEG200 did not affect rotation, but, as previously reported, PEG8000 inhibited rotation [3] (figure 3*a–d*). This suggests that inhibition of T3SA rotation by PEG8000 is attributable to the viscosity of the PEG8000 solution. We also explored the suppression of effector secretion under these conditions. PEG200 did not affect secretion, although PEG8000 suppressed secretion in the manner described for PAO1 WT (figure 3*e*). The suppressive effect of PEG8000 was enhanced in the presence of microbeads, as previously reported [3]. The data therefore suggest that PEG8000 inhibits T3SA rotation by increasing medium viscosity and thus suppresses effector secretion.

### 4.4. Suppression of effector secretion by alginate

**Figure 4.**
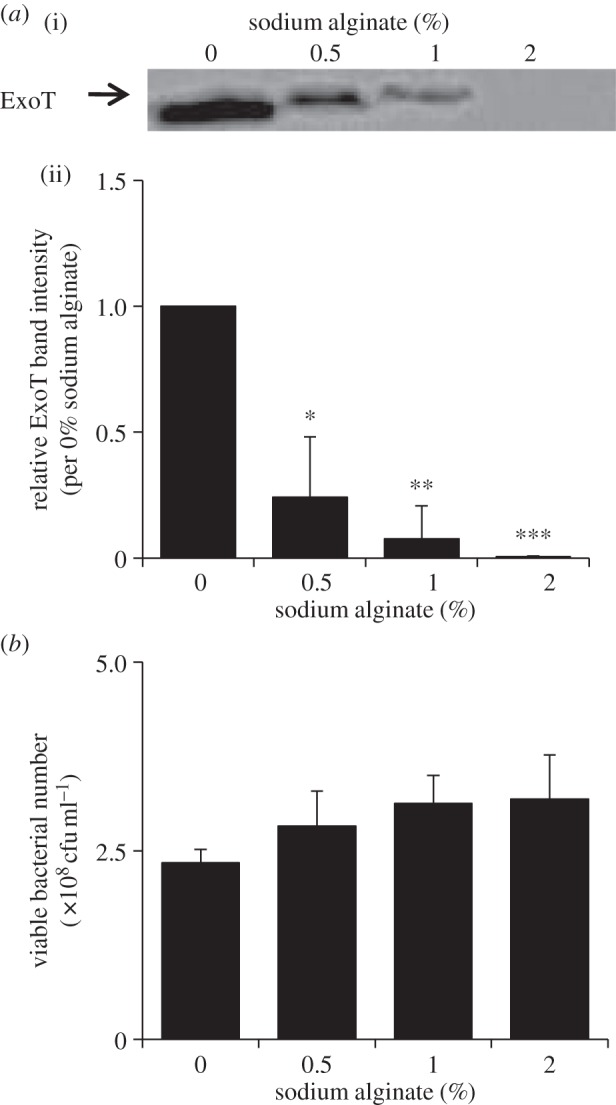
Effect of sodium alginate on ExoT secretion by WT PAO1. (*a*) Western blot analysis of ExoT protein levels in supernatants of WT PAO1 cultured in the presence of various concentrations of sodium alginate. Data were collected and analysed as described in figure 1*a*. Average values ± s.d. (*n* = 3) are shown. *p < 0.05, **p < 0.01, ***p < 0.001. (*b*) Effect of sodium alginate on bacterial viability quantified by colony counting. Average values ± s.d. (*n* = 3) are shown. No significant differences were seen in bacterial viability between 0% and 0.5–2% alginate-added samples (*p* < 0.05 versus 0% alginate).

**Figure 5.**
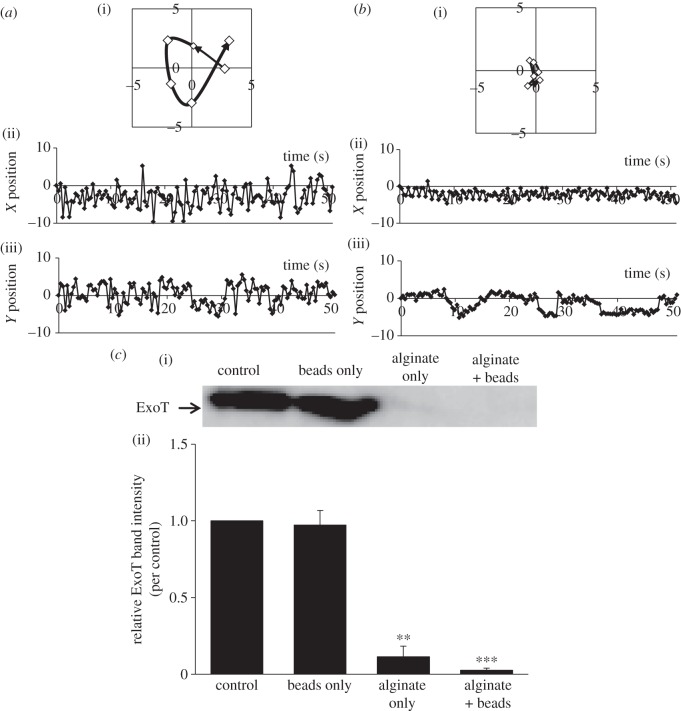
Effect of sodium alginate on T3SA rotation and effector secretion by PAO1 Strep. (*a*,*b*) Analysis of T3SA rotation in PAO1 Strep (*a*) before and (*b*) after treatment with 1% (w/v) alginate. Data were collected and analysed as described in figure 3*a–d*. (*c*) Western blotting analysis of ExoT protein levels in the supernatants of cultures of PAO1 Strep grown in the presence of 1% (w/v) alginate and T3SA-binding microbeads. Data were collected and analysed as described in figure 3*e*. Average values ± s.d. (*n* = 3) are shown. **p < 0.01, ***p < 0.001.

We hypothesized that viscous polymers other than PEG8000 would also suppress secretion through T3SA, if the viscosity of PEG8000 mainly contributes to the suppression. Therefore, we explored the effect of alginate on effector secretion. Alginate solution (like PEG8000 solution) is of high viscosity (see electronic supplementary material, table S1), but alginate is negatively charged (PEG8000 is neutral) and alginate is formed from components (mannuronic and guluronic acid) that differ from the PEG8000 monomer (ethylene glycol). Alginate suppressed effector secretion by PAO1 WT significantly, as did PEG8000 (figure 4), and also inhibited both T3SA rotation and effector secretion by PAO1 Strep significantly, as did PEG8000 (figure 5). Therefore, it was suggested that the inhibitory effect of effector secretion and T3SA rotation is not specific for PEG8000, but other viscous polymers could show the same effect.

### 4.5. Correlations between solution viscosity and suppression of effector secretion by polymers

**Figure 6.**
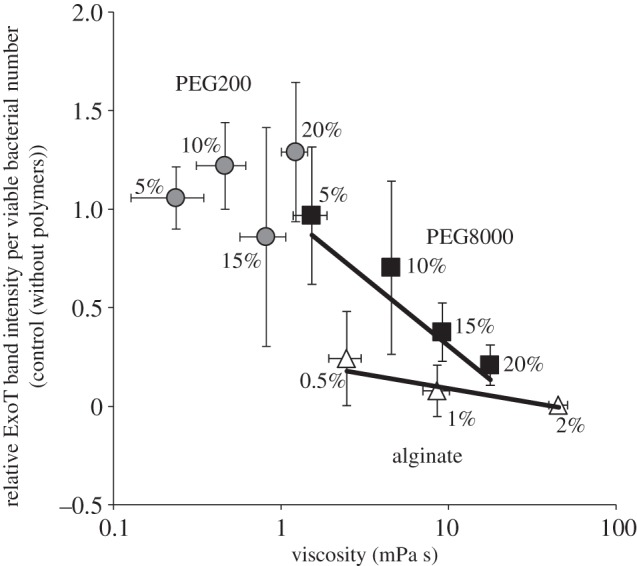
Association between polymer viscosity and suppression of effector secretion. Polymer viscosity influences effector secretion. The correlation curve of PEG200, PEG8000 and alginate between viscosity and relative ExoT band intensity per viable bacterial number are shown. The ExoT band intensity per viable bacterial number without any polymers was set to 1.0. The values used to construct the figure are shown in the electronic supplementary material, table S1. Average values ± s.d. (*n* = 3) are shown.

To evaluate the contribution of polymer viscosity to suppression of effector secretion in more detail, we measured the viscosities of various polymer solutions and sought correlations between these values and suppression of secretion. The viscosity of PEG8000 solutions and the suppressive effects of such solutions on effector secretion were highly correlated (correlation coefficient = −0.944; figure 6). PEG200 solutions were of very low viscosity; the viscosity of 20% (v/v) PEG200 was lower than that of 5% (w/v) PEG8000 (see electronic supplementary material, table S1). These results suggest that medium viscosity is an important contributor to the suppression of effector secretion. The viscosities of alginate solutions also correlated highly with the suppression of effector secretion (correlation coefficient = −0.819; figure 6). However, the dependence of suppression efficiency on the viscosity of alginate solutions was lower than that shown by PEG8000 (the slopes of the correlation diagrams were −0.004 and −0.045, respectively). This suggests that, in the case of alginate, not only viscosity but also other factors (e.g. electrostatic interactions) may contribute to the observed high-level suppression of effector secretion.

### 4.6. Suppression of effector secretion by T3SA using the natural polymer mucin

**Figure 7.**
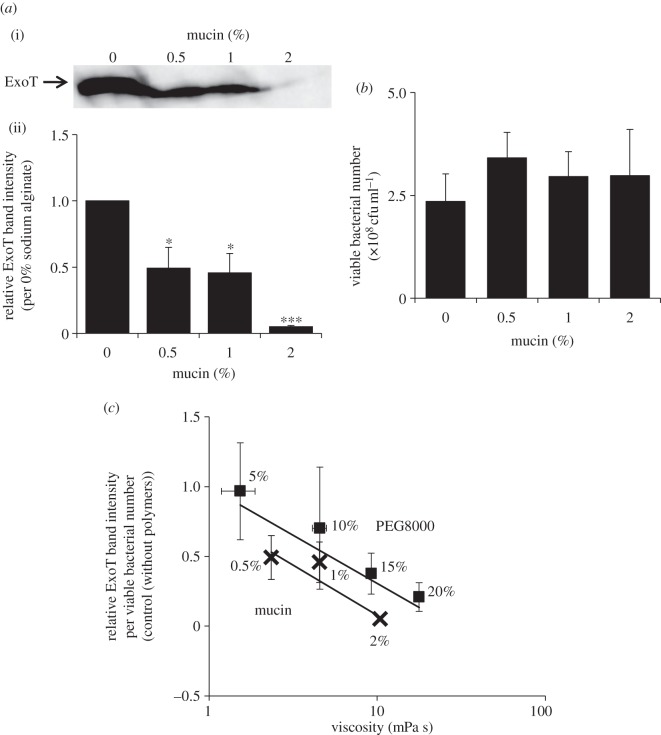
Effect of mucin on ExoT secretion by WT PAO1. (*a*) Western blotting analysis of ExoT protein levels in supernatants of WT PAO1 cultured in the presence of different concentrations of mucin. Data were collected and analysed as described in figure 1*a*. Average values ± s.d. (*n* = 3) are shown. *p < 0.05, ***p < 0.001. (*b*) Effect of mucin on bacterial viability measured by colony counting. Average values ± s.d. (*n* = 3) are shown. No significant differences were seen in bacterial viability between 0% and 0.5–2% mucin added samples (*p* < 0.05 versus 0% mucin). (*c*) The correlation curve of PEG8000 and mucin between the viscosity and suppression of effector secretion. The graph was plotted as described in figure 6. Average values ± s.d. (*n* = 3) are shown.

**Figure 8.**
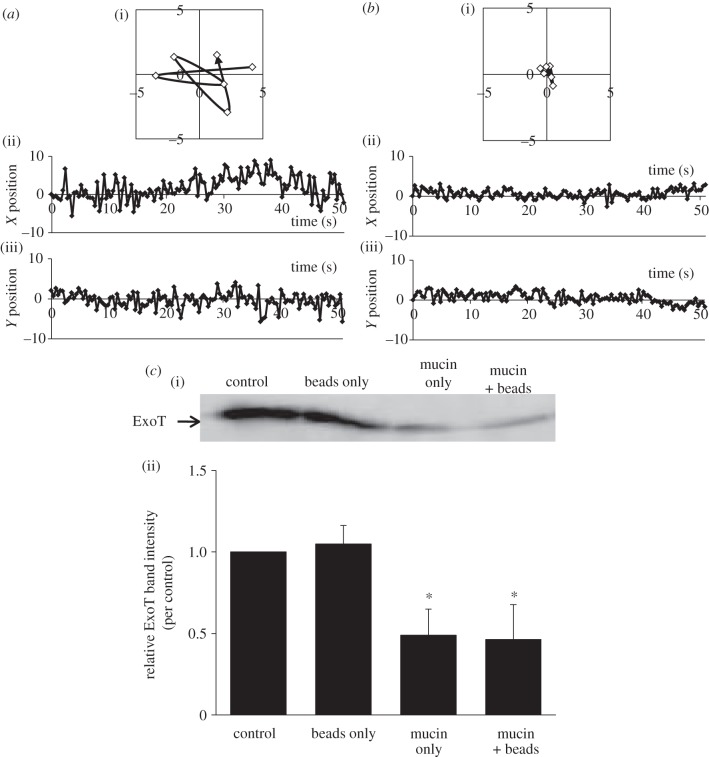
Effect of mucin on T3SA rotation and effector secretion by PAO1 Strep. (*a*,*b*) T3SA rotation (*a*) before and (*b*) after treatment with 1% (w/v) mucin. The data were collected and analysed as described in figure 3*a*–*d*. (*c*) Western blotting analysis of ExoT protein levels in supernatants of PAO1 Strep cultured in the presence of 1% (w/v) mucin and microbeads. Data were collected and analysed as described in figure 3*e*. Average values ± s.d. (*n* = 3) are shown. *p < 0.05.

Alginate is a natural polymer produced by *P. aeruginosa* to form biofilms [11]. The exact amount of alginate in biofilm is unknown, because it varies significantly according to environmental changes. It was reported that 1% (w/v) concentration of alginate added to culture of non-mucoid PAO1 strain behaved similarly to that produced naturally by mucoid FRD1 strain, isolated from a cystic fibrosis patient [12]. Thus, the work described above might reflect the natural biofilm environment and suggests that regulation of effector secretion by polymers may occur in naturally viscous environments. We next asked whether another natural polymer, mucin, might affect secretion. It was reported that the concentration of mucin *in vivo* is higher than 2% (w/v) [13]. Therefore, we used mucin at 0–2% (w/v) concentration in our experiment. Mucin suppressed effector secretion by PAO1 WT in a manner similar to that of PEG8000 and alginate (figure 7). The suppression of mucin on effector secretion also correlate highly to its viscosity (correlation coefficient = −0.981; figure 7*c*). Mucin also inhibited both the rotation of and secretion through T3SA in PAO1 Strep (figure 8). These results suggest that mucin might also suppress the effector secretion via inhibition of T3SA rotation. Mucin, produced by epithelial cells, has a negatively charged domain [14,15], as does alginate, and functions as a layer of defence against bacterial infection [16]. Thus, T3SA rotation and effector secretion may be suppressed by natural polymers, such as alginate and mucin, in naturally viscous environments including biofilms and mucin layers.

## 5. Discussion

In this study, we examined the suppressive effects of various polymers on effector secretion to determine the relevant modes of action.

The suppressing effect of PEG8000 on effector secretion would not depend on the transcriptional suppression of effector genes, because PEG8000 was observed to activate transcriptional expression rather than suppress it (see electronic supplementary material, figure S1). The comparative data on PEG8000 and PEG200 suggest that the suppression of effector secretion by PEG8000 is independent of properties intrinsic to the monomeric component, ethylene glycol (figure 2); it is associated with the inhibition of T3SA rotation (figure 3). We found that the viscosity of PEG8000 solutions correlated highly with the suppression of effector secretion. PEG200, which did not affect T3SA rotation or secretion, formed solutions of low viscosity (figure 6). Thus, medium viscosity plays a major role in the suppression of effector secretion by polymers. It has been reported that increasing viscous resistance inhibits the motility of molecular motors (of the flagellum or F1-ATPase) and suppresses functions [4,5]. Therefore, our results strongly support the notion that PEG8000 suppresses effector secretion by T3SA via physico-chemical inhibition of T3SA rotation caused by the high viscosity of PEG8000 solutions.

This possibility was supported by the finding that alginate solutions, which are also highly viscous, inhibited both T3SA rotation and effector secretion (figures 4 and 5), and suppression of secretion was well correlated with solution viscosity (figure 6); alginate monomers differ from that of PEG8000 [17].

Inhibition of effector diffusion from T3SA needle is also a possible factor influencing the suppression of secretion by polymers. At this time, it is difficult to ascertain the contribution, but this would not be the only factor contributing to the suppression. According to Fick's second law (*∂c*/*∂t* = *D*(∂^2^*c*/*∂x*^2^), *D* = *k_B_T*/6*π*r*η*), polymers might affect effector diffusion only by increasing viscosity, regardless of their components. However, the dependence of suppression level on alginate viscosity differed from that of PEG8000 (figure 6). This result suggests that the suppressing effect on effector secretion is influenced not only by polymer viscosity, but also by their components. Therefore, inhibition of effector diffusion from T3SA needle is at least not the only factor contributing to the suppression of effector secretion. The two polymers differ in electrostatic properties (alginate and PEG8000 are negatively charged and neutral, respectively) [17]. Recently, it has been reported that the N-terminal domains of T3SA needle components are located on the exterior surface of the needle [18]. Therefore, this may be because alginate interacts with T3SA better than does PEG8000 via the electrostatic interaction with the N-terminal amino groups. This result suggests that high viscosity is not the only factor contributing to suppression of effector secretion; other factors (e.g. electrostatic interaction between a polymer and T3SA) may also play a role.

By contrast, the addition of microbeads alone did not affect the secretion, although it would certainly increase the rotational resistance (figure 3*e*). This might suggest the existence of a threshold of rotational resistance for the suppression of effector secretion. Rotational resistance by modifying T3SA needle with microbeads might be insufficient to suppress secretion. This speculation is supported by the observation that simultaneous addition of PEG8000 and beads suppressed effector secretion more effectively than individual addition (figure 3*e*). In this case, by adding PEG8000 simultaneously with beads, the resistance might overcome the threshold and, as a result, the effector secretion would be suppressed. To evaluate the relationship between T3SA rotation and effector secretion in greater depth, simultaneous evaluation of T3SA rotation speed and effector secretion rate is required. In the future, this may be achieved by improving observation of T3SA rotation and development of real-time measures of effector secretion. In any case, it is likely that inhibition of T3SA rotation contributes to the suppression of effector secretion, because this was true when both PEG8000 and alginate were tested (figure 5).

Moreover, the alginate data suggest that not only artificial, but also natural polymers may suppress effector secretion; alginate is the major component of biofilms formed by *P. aeruginosa.* Mucin, a major component of the mucin layers produced by epithelial cells to defend against the bacterial (e.g. *P. aeruginosa*) infection [15], also suppressed effector secretion (figure 7). Mucin also inhibited T3SA rotation when effector secretion was suppressed (figure 8). The suppressing rate of mucin on secretion correlated highly with its viscosity, similar to PEG8000 or alginate (figure 7*c*). The suppression efficiency was slightly higher than that of PEG8000, but lower than that of alginate at the same viscosity (the slope of the correlation diagrams of mucin solution was −0.057; figures 6 and 7*c*). This result might support the importance of electrostatic interaction with T3SA needle on suppression of effector secretion. Although mucin has a negatively charged domain as part of its structure [14], the charge density would be lower than that of alginate (which is negatively charged all over the structure) [11]. Therefore, it might interact with T3SA needle more strongly than PEG8000 (its charge is neutral), but less strongly than alginate. The results shown in figures 6 and 7*c* might reflect this tendency. These results suggest that effector secretion may be naturally regulated via physico-chemical inhibition of T3SA rotation by natural polymers in viscous environments, such as biofilms and mucin layers.

The effector secretion of T3SA requires both ATP and the proton-motive force [19,20]. Effector secretion by *P. aeruginosa* is induced by removal of Ca^2+^ by EGTA [21], and alginate also chelates Ca^2+^ [22]. Therefore, secretion would be induced in a biofilm if the only regulatory system in play was the Ca^2+^-dependent system derived above. However, secretion would not be necessary, because (at least) all known effectors perform no essential activities in biofilms [2,23] and deletion of T3SA upregulates biofilm formation [24,25]. Indeed, induction of secretion in biofilms would often be a crucial waste of energy. The efficiency of ATP production by bacteria located deep within biofilms is low because the environment is anaerobic and nutrient production is low [26–28]. Therefore, it would be reasonable to inhibit effector secretion by biofilm bacteria to avoid wastage of ATP. Similarly, effector secretion by bacteria in mucin layers is not required but flagellar rotation should be enhanced to allow passage through such layers [29,30]. It is thus reasonable that, in a mucin layer, the proton-motive force normally required for T3SA action is rather used exclusively by the flagellum.

Suppression of unnecessary secretion by natural environmental polymers, such as biofilms or mucin layers, may function to minimize energy wastage. Moreover, such systems would allow conservation of effectors; the materials would be secreted only when appropriate. Such systems could respond quickly to environmental changes, because the trigger for action is not complex; the only step required is physico-chemical inhibition of T3SA rotation. Therefore, it is very possible that bacteria use such regulatory systems to control effector secretion, although direct evidence for this suggestion remains to be gathered.

In conclusion, we have confirmed that high medium viscosity induced by dissolved polymers suppresses effector secretion. However, not only viscosity but also other features of polymers (e.g. a capacity for electrostatic interaction) may also contribute to the observed effects. Moreover, the work suggests that the suppression of effector secretion by polymers may occur naturally in biofilms and mucin layers. We expect that our findings will contribute to an elucidation of how effector secretion is regulated.

## Supplementary Material

Supplemental Figure 1; Supplemental Figure 2; Supplemental Figure 3

## Supplementary Material

Supplemental Table 1

## References

[1] Cornelis GR 2006 The type III secretion injectisome. Nat. Rev. Microbiol. 4, 811–825 (doi:10.1038/nrmicro1526) 17041629 10.1038/nrmicro1526

[2] Engel J Balachandran P 2009 Role of *Pseudonomas aeruginosa* type III effectors in disease. Curr. Opin. Microbiol. 12, 61–66 (doi:10.1016/j.mib.2008.12.007) 19168385 10.1016/j.mib.2008.12.007

[3] Ohgita T Hayashi N Hama S Tsuchiya H Gotoh N Kogure K 2013 A novel effector secretion mechanism based on proton-motive force-dependent type III secretion apparatus rotation. FASEB J. 27, 2862–2872 (doi:10.1096/fj.13-229054) 23515444 10.1096/fj.13-229054

[4] Magariyama Y Sugiyama S Kudo S 2001 Bacterial swimming speed and rotation rate of bundled flagella. FEMS Microbiol. Lett. 199, 125–129 (doi:10.1111/j.1574-6968.2001.tb10662.x) 11356579 10.1111/j.1574-6968.2001.tb10662.x

[5] Spetzler D Ishmukhametov R Hornung T Day LJ Martin J Frasch WD 2009 Single molecule measurements of F1-ATPase reveal an interdependence between the power stroke and the dwell duration. Biochemistry 48, 7979–7985 (doi:10.1021/bi9008215) 19610671 10.1021/bi9008215 PMC2737049

[6] McNamee CE Pyo N Higashitani K 2006 Atomic force microscopy study of the specific adhesion between a colloid particle and a living melanoma cell: effect of the charge and the hydrophobicity of the particle surface. Biophys. J. 91, 1960–1969 (doi:10.1529/biophysj.106.082420) 16731555 10.1529/biophysj.106.082420 PMC1544312

[7] McNamee CE Yamamoto S Higashitani K 2007 Effect of the physicochemical properties of polyethylene glycol brushes on their binding to cells. Biophys. J. 93, 324–334 (doi:10.1529/biophysj.106.102251) 17434943 10.1529/biophysj.106.102251 PMC1914419

[8] Stover CK 2000 Complete genome sequence of *Pseudomonas aeruginosa* PAO1, an opportunistic pathogen. Nature 406, 959–964 (doi:10.1038/35023079) 10984043 10.1038/35023079

[9] Lee PC Stopford CM Svenson AG Rietsch A 2010 Control of effector export by the *Pseudomonas aeruginosa* type III secretion proteins PcrG and PcrV. Mol. Microbiol. 75, 924–941 (doi:10.1111/j.1365-2958.2009.07027.x) 20487288 10.1111/j.1365-2958.2009.07027.x PMC3124366

[10] Okuda J Okamoto M Hayashi N Sawada S Minagawa S Gotoh N 2012 Complementation of the exoS gene in the pvdE pyoverdine synthesis gene-deficient mutant of *Pseudomonas aeruginosa* results in recovery of the pvdE gene-mediated penetration through the intestinal epithelial cell barrier but not the pvdE-mediated virulence in silkworms. J. Infect. Chemother. 18, 332–340 (doi:10.1007/s10156-011-0340-0) 22080193 10.1007/s10156-011-0340-0

[11] Boyd A Chakrabarty AM 1995 *Pseudomonas aeruginosa* biofilms: role of the alginate exopolysaccharide. J. Ind. Microbiol. 15, 162–168 (doi:10.1007/BF01569821) 8519473 10.1007/BF01569821

[12] Horsman SR Moore RA Lewenza S 2012 Calcium chelation by alginate activates the type III secretion system in mucoid *Pseudomonas aeruginosa* biofilm. PLoS ONE 7, e46826 (doi:10.1371/journal.pone.0046826) 23056471 10.1371/journal.pone.0046826 PMC3466208

[13] Bansil R Stanley E LaMont JT 1995 Mucin biophysics. Annu. Rev. Physiol. 57, 635–657 (doi:10.1146/annurev.ph.57.030195.003223) 7778881 10.1146/annurev.ph.57.030195.003223

[14] Bansil R Turner BS 2006 Mucin structure, aggregation, physiological functions and biomedical applications. Curr. Opin. Colloid Interface Sci. 11, 164–170 (doi:10.1016/j.cocis.2005.11.001)

[15] Sandberg T Blom H Caldwell KD 2009 Potential use of mucins as biomaterial coatings. I. Fractionation, characterization, and model absorption of bovine, porcine, and human mucins. J. Biomed. Mater. Res. A 91, 762–772 (doi:10.1002/jbm.a.32266) 19051309 10.1002/jbm.a.32266

[16] McGuckin MA Lindén SK Sutton P Florin TH 2011 Mucin dynamics and enteric pathogens. Nat. Rev. Microbiol. 9, 265–278 (doi:10.1038/nrmicro2538) 21407243 10.1038/nrmicro2538

[17] Drury JL Mooney DJ 2003 Hydrogels for tissue engineering: scaffold design variables and applications. Biomaterials 24, 4337–4351 (doi:10.1016/S0142-9612(03)00340-5) 12922147 10.1016/s0142-9612(03)00340-5

[18] Lonquet A 2012 Atomic model of the type III secretion system needle. Nature 486, 276–279 (doi:10.1038/nature11079) 22699623 10.1038/nature11079 PMC3598588

[19] Akeda Y Galán JE 2005 Chaperone release and unfolding of substrates in type III secretion. Nature 437, 911–915 (doi:10.1038/nature03992) 16208377 10.1038/nature03992

[20] Wilharm G Lehmann V Krauss K Lehnert B Richter S Ruckdeschei K Heesemann J Trülzsch K 2004 Yersinia enterocolitica type III secretion depends on the proton motive force but not on the flagellar motor components MotA and MotB. Infect. Immun. 72, 4004–4009 (doi:10.1128/IAI.72.7.4004-4009.2004) 15213145 10.1128/IAI.72.7.4004-4009.2004 PMC427454

[21] Kim J Ahn K Min S Jia J Ha U Wu D Jin S 2005 Factors triggering type III secretion in *Pseudomonas aeruginosa*. Microbiology 151, 3575–3587 (doi:10.1099/mic.0.28277-0) 16272380 10.1099/mic.0.28277-0

[22] Lattner D Flemming HC Mayer C 2003 13C-NMR study of the interaction of bacterial alginate with bivalent cations. Int. J. Biol. Macromol. 33, 81–88 (doi:10.1016/S0141-8130(03)00070-9) 14599588 10.1016/s0141-8130(03)00070-9

[23] Hauser AR 2009 The type III secretion system of *Pseudomonas aeruginosa*: infection by injection. Nat. Rev. Microbiol. 7, 654–665 (doi:10.1038/nrmicro2199) 19680249 10.1038/nrmicro2199 PMC2766515

[24] Rawls JF Mahowald MA Goodman AL Trent CM Gordon JI 2007 In vivo imaging and genetic analysis link bacterial motility and symbiosis in the zebrafish gut. Proc. Natl Acad. Sci. USA 104, 7622–7627 (doi:10.1073/pnas.0702386104) 17456593 10.1073/pnas.0702386104 PMC1855277

[25] Goodman AL Kulasekara B Rietsch A Boyd D Smith RS Lory S 2004 A signaling network reciprocally regulates genes associated with acute infection and chronic persistence in *Pseudomonas aeruginosa**.* Dev. Cell 7, 745–754 (doi:10.1016/j.devcel.2004.08.020) 15525535 10.1016/j.devcel.2004.08.020

[26] Hall-Stoodley L Costerton JW Stoodley P 2004 Bacterial biofilms: from the natural environment to infectious diseases. Nat. Rev. Microbiol. 2, 95–108 (doi:10.1038/nrmicro821) 15040259 10.1038/nrmicro821

[27] Yoon SS 2002 *Pseudomonas aeruginosa* anaerobic respiration in biofilms: relationships to cystic fibrosis pathogenesis. Dev. Cell 3, 593–603 (doi:10.1016/S1534-5807(02)00295-2) 12408810 10.1016/s1534-5807(02)00295-2

[28] Nadell CD Xavier JB Foster KR 2009 The sociobiology of biofilms. FEMS Microbiol. Rev. 33, 206–224 (doi:10.1111/j.1574-6976.2008.00150.x) 19067751 10.1111/j.1574-6976.2008.00150.x

[29] Liu Z Miyashiro T Tsou A Hsiao A Goulian M Zhu J 2008 Mucosal penetration primes *Vibrio cholera* for host colonization by repressing quorum sensing. Proc. Natl Acad. Sci. USA 105, 9769–9774 (doi:10.1073/pnas.0802241105) 18606988 10.1073/pnas.0802241105 PMC2474479

[30] Hayashi N Matsukawa M Horinishi Y Nakai K Shoji A Yoneko Y Yoshida N Minagawa S Gotoh N 2013 Interplay of flagellar motility and mucin degradation stimulates the association of *Pseudomonas aeruginosa* with human epithelial colorectal adenocarcinoma (Caco-2) cells. J. Infect. Chemother. 19, 305–315 (doi:10.1007/s10156-013-0554-4) 23340990 10.1007/s10156-013-0554-4

