# Supplementary figures and images for: Suppression of type III effector secretion by polymers
Source: Open Biol. 2013 Dec;3(12):130133. doi: 10.1098/rsob.130133 (PMC3877841; doi:10.1098/rsob.130133)

Supplemental Figure S1

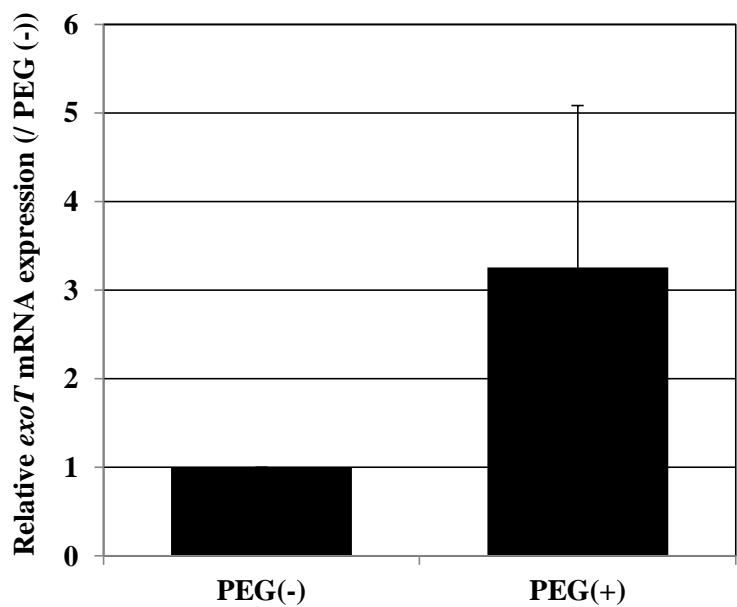

**Supplemental Figure S2**

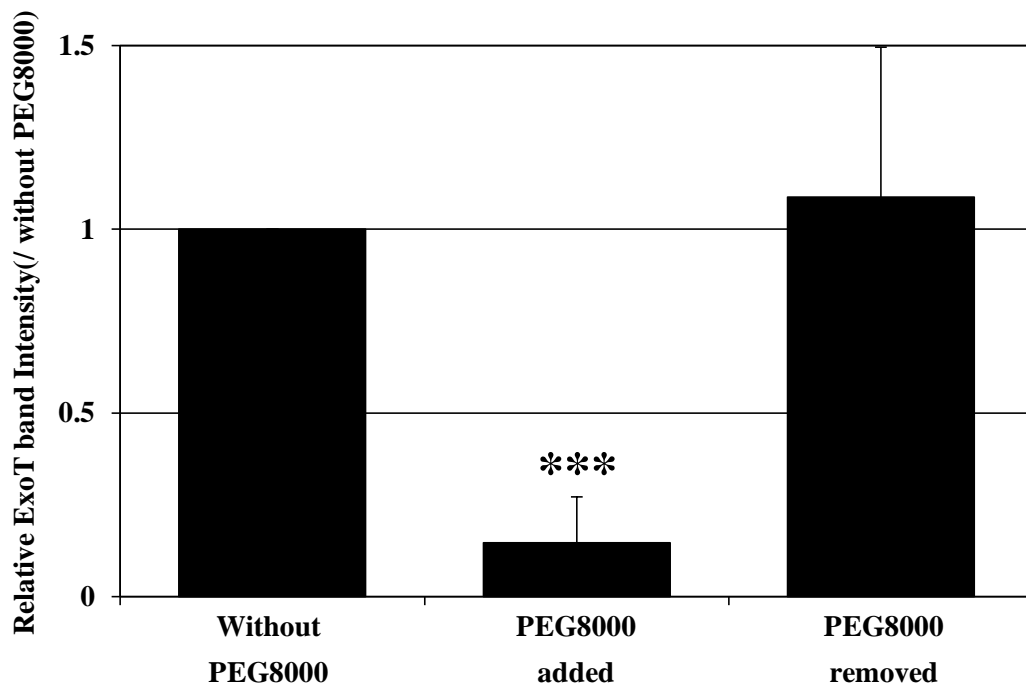

Supplemental Figure S3

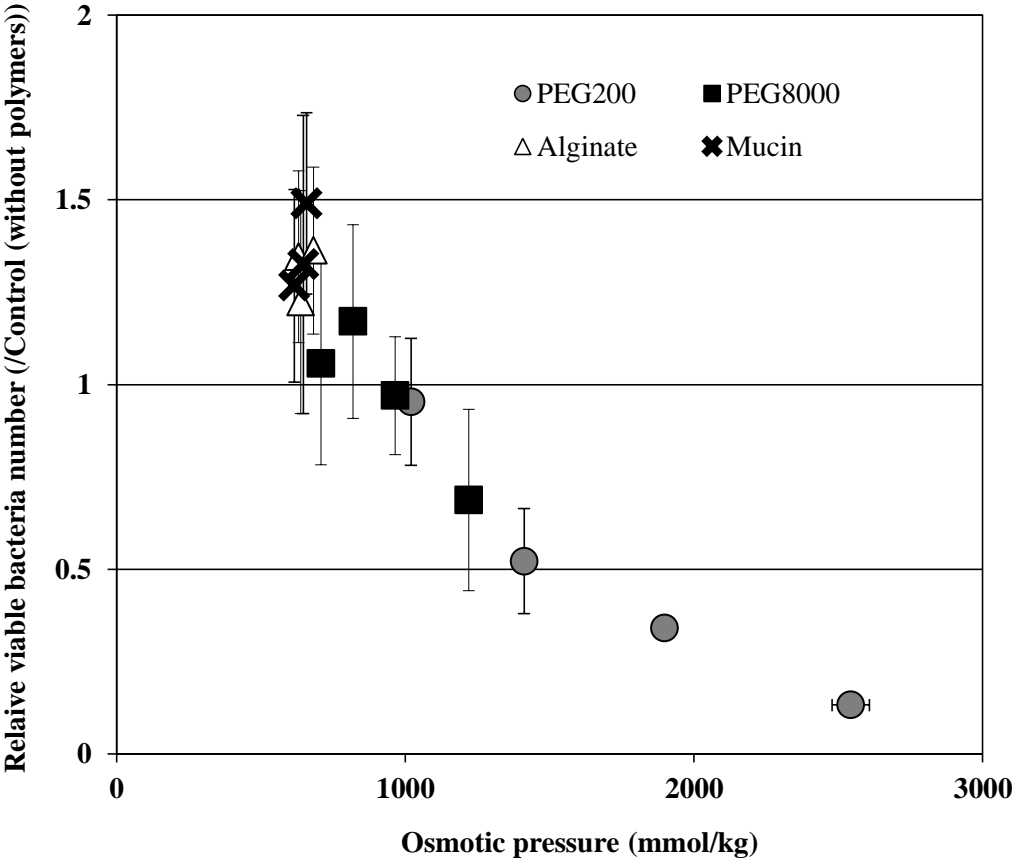

Supplement: Supplemental Figure 1; Supplemental Figure 2; Supplemental Figure 3 [file rsob130133supp1.pdf]
