# Supplementary material for: Suppression of type III effector secretion by polymers
Source: Open Biol. 2013 Dec;3(12):130133. doi: 10.1098/rsob.130133 (PMC3877841; doi:10.1098/rsob.130133)
Supplement: Supplemental Table 1 [file rsob130133supp2.pdf]

Supplemental Table S1

PEG8000 (%)	Viscosity (mPa·s)	Relative ExoT band intensity per viable bacterial number (/ Control (without polymers))	Osmotic pressure (mmol/kg)	Relative viable bacterial number (/ Control (without polymers))
5	1.53±0.18	0.97±0.35	708±19.1	1.06±0.27
10	4.56±0.41	0.70±0.44	819±12.8	1.17±0.26
15	9.19±0.82	0.37±0.15	965±12.0	0.97±0.16
20	17.7±2.05	0.20±0.10	1221±27.3	0.68±0.25

PEG200 (%)	Viscosity (mPa·s)	Relative ExoT band intensity per viable bacterial number (/ Control (without polymers))	Osmotic pressure (mmol/kg)	Relative viable bacterial number (/ Control (without polymers))
5	0.24±0.11	1.05±0.16	1020±7.78	0.95±0.17
10	0.46±0.15	1.22±0.22	1412±9.53	0.52±0.14
15	0.82±0.25	0.85±0.56	1899±39.4	0.34±0.02
20	1.22±0.22	1.29±0.35	2543±64.7	0.13±0.011

Alginate (%)	Viscosity (mPa·s)	Relative ExoT band intensity per viable bacterial number (/ Control (without polymers))	Osmotic pressure (mmol/kg)	Relative viable bacterial number (/ Control (without polymers))
0.5	2.47±0.54	0.24±0.24	638±15.9	1.22±0.30
1	8.56±1.54	0.07±0.13	631±17.1	1.35±0.23
2	45.5±5.70	0.005±0.0031	682±1.26	1.36±0.23

Mucin (%)	Viscosity (mPa·s)	Relative ExoT band intensity per viable bacterial number (/ Control (without polymers))	Osmotic pressure (mmol/kg)	Relative viable bacterial number (/ Control (without polymers))
0.5	1.45±0.04	0.49±0.15	659±0.58	1.49±0.25
1	3.64±0.09	0.46±0.14	647±1.53	1.33±0.40
2	9.51±0.17	0.05±0.0086	616±1.00	1.27±0.26
